# Contemporary Methods for Detection and Intervention of Distal Medium and Small Vessel Occlusions

**DOI:** 10.3390/jcm12186071

**Published:** 2023-09-20

**Authors:** Anthony Piscopo, Mario Zanaty, Kathleen Dlouhy

**Affiliations:** Department of Neurosurgery, University of Iowa Hospital and Clinics, Iowa City, IA 52242, USA; anthony-piscopo@uiowa.edu (A.P.); kathleen-dlouhy@uiowa.edu (K.D.)

**Keywords:** mechanical thrombectomy, stroke, endovascular, imaging, detection, intervention

## Abstract

The efficacy of using mechanical thrombectomy for proximal large vessel occlusions has been demonstrated in multiple large-scale trials and has further raised the question of its potential utility for distal medium and small vessel occlusions (DMSVOs). Their longer, more tortuous course and smaller corresponding vascular territories render a significant challenge for detection and intervention. The aim of this study is to provide a comprehensive overview of the current imaging and endovascular intervention options for DMSVOs and review the current works in the literature. Compared with traditional computed tomography angiography (CTA) and CT perfusion, recent advances such as multiphase CTA and maps derived from the time-to-maximum parameter coupled with artificial intelligence have demonstrated increased sensitivity for the detection of DMSVOs. Furthermore, newer generations of mini stent retrievers and thromboaspiration devices have allowed for the access and navigation of smaller and more fragile distal arteries. Preliminary studies have suggested that mechanical thrombectomy using this newer generation of devices is both safe and feasible in distal medium-sized vessels, such as M2. However, endovascular intervention utilizing such contemporary methods and devices must be balanced at the discretion of operator experience and favorable vascular anatomy. Further large-scale multicenter clinical trials are warranted to elucidate the indications for as well as to strengthen the safety and efficacy of this approach.

## 1. Introduction

In the setting of large vessel acute ischemic stroke, mechanical thrombectomy (MT) has been established as a gold-standard treatment both within 6 h and up to 24 h from symptom onset in various clinical trials [1,2]. In this setting, MT acts to restore cerebral perfusion and has led to improved clinical outcomes as well as increased functional independence and lower rates of disability when compared with standard care alone [3,4].

The success of MT in proximal large vessel occlusion (PLVO) has raised the question of its safety, feasibility, and efficacy in cases of distal medium (DMSVO) and small vessel occlusion (SVO) as well. Compared with PLVOs (intracranial ICA, M1 MCA segment, intracranial vertebral arteries, and basilar artery), occlusions in more distal, medium-sized, and small-sized vessels present several challenges for endovascular intervention. The primary considerations include a smaller vessel diameter, a longer route, and increased tortuosity and friability. Thus, during manipulation and therapeutic intervention, caution must be taken regarding the risks of dissection, vessel rupture, and subsequent vasospasm [5,6].

Although presentations of acute ischemic stroke for thrombectomy are most commonly due to PLVOs, it is estimated that 25 to 40% of cases are due to DMSVOs and 20 to 25% of cases are due to SVOs of deep penetrating arteries [7,8]. DMSVOs may arise de novo or secondary to fragmentation and embolization during endovascular thrombectomy (EVT) for PLVO, and they were shown to occur in as many as 14% of cases [9]. Despite the demonstrated benefit of using EVT for eligible patients with acute ischemic stroke within 6 h of symptom onset, there have been no dedicated clinical trials investigating the utility of EVT in cases of DMSVO and SVO [10].

Multicenter studies have demonstrated that DMSVOs treated medically, particularly in M2, are associated with high rates of functional dependency, neurological deficit, and mortality [11]. Both primary and secondary DMSVOs may lead to motor, sensory, and cognitive deficits as well as a risk of death. However, secondary DMSVOs are often associated with more severe clinical presentation and a larger magnitude of neurological deficit, as they typically result from more proximal occlusions and larger areas of infarct [12].

There have been significant advances in the diagnosis and intervention of DMSVOs and SVOs in recent years [13,14,15]. While current imaging modalities are both sensitive and specific for PLVOs, more distal occlusions are often missed in the initial diagnostic workup in as many as 82% of cases as reported in the literature, rendering patients susceptible to further neurological damage [16]. The implementation of contemporary imaging techniques including multiphase computed tomography angiography (CTA), deep learning and artificial intelligence, and time-to-maximum tissue residue function maps have been shown to accurately detect PLVO in several studies and are an exciting avenue of active investigation for DMSVOs and SVOs [17,18]. Additionally, new devices for MT and thromboaspiration have been shown to safely access and treat DMSVOs, and provide advantages including having a small size and an increased maneuverability, allowing for the treatment of vessels that were previously inaccessible when using traditional methods [13,19]. The objectives of the current study are to provide a description and overview of modern imaging and endovascular intervention methods for the evaluation and intervention of DMSVOs as well as to review the current literature.

## 2. Defining the Segments

PLVOs are classified as occlusions of the ICA, M1 MCA segment, vertebral arteries, and basilar artery in a widely agreed upon classification. Medium-sized vessels, which are technically defined as cerebral vessels with lumen diameters of 0.75 to 2.0 mm, have seen several classification schemas with controversy surrounding the grouping of M2 segments, which range from 1.1 to 2.1 mm. However, an international consensus group most recently proposed the terms DMSVO and PLVO, which, in contrast to the previous terms “medium vessel occlusion” and “large vessel occlusion”, consider both the distance tortuosity and size. DMSVOs include the M2–M4 middle cerebral artery (MCA), the M2 non-dominant trunk, the anterior cerebral artery (ACA), the anterior inferior cerebellar artery (AICA), the posterior inferior cerebellar artery (PICA), the superior cerebellar artery (SCA), and the posterior cerebral artery (PCA) [20].

## 3. Methodology and Search Strategy

A comprehensive literature review was performed using the Pubmed database with keywords of variable combinations including “vessel occlusion”, “distal”, “medium”, “thrombolysis”, “endovascular”, “imaging”, “detection”, “mechanical thrombectomy”, and “thromboaspiration”. Peer-reviewed studies evaluating mechanical thrombectomy, thromboaspiration, intravascular thrombolysis, or imaging/detection methods for DMSVO were included. Studies strictly evaluating PLVO were excluded. The NIH clinicaltrials.gov registry was also queried for randomized controlled trials currently ongoing and/or enrolling patients for DMSVO. Recent randomized controlled trials and guidelines from the European Stroke Organisation were considered for treatment recommendations [21].

## 4. Imaging

The standard-of-care for the rapid diagnosis of AIS includes non-contrast head computed tomography (CT) followed by CT angiography (CTA) [22]. Additionally, CT perfusion (CTP) provides measures of the cerebral blood flow and cerebral blood volume, allowing for the estimation of at-risk but still viable brain tissue (i.e., penumbra) [23]. Magnetic resonance imaging (MRI) may also be utilized, particularly in cases of an unknown time of onset, but has not been as widely adopted in the acute setting [24]. However, a comparison of CT with MRI in 401 cases of AIS showed that the average stroke-onset-to-imaging time was only 7 min longer for MRI than CT (114 vs. 107 min, respectively) [25].

CTP has become a mainstay of imaging protocols for AIS when available. Its color-coded visuospatial display allows for the accurate determination of the location and penumbra in AIS using mean transit time, cerebral blood flow, and cerebral blood volume, with a minimal learning curve for interpretation [26].

Despite the sensitivity, specificity, and widespread adoption of CT and CTP in AIS, they are not without drawbacks. Cost, availability, the need for additional radiation, susceptibility to motion and flow artifact, a lack of whole-brain coverage, and the need for postprocessing software relying on algorithms and thresholds for predicting core infarct that are yet to be agreed upon by experts are all significant factors associated with CTP that need to be considered in AIS [27,28]. Additionally, interpretation errors and interrater discrepancies are not uncommon in CT imaging, with error rates between 3 and 5% being commonly reported, with some studies suggesting as high as 13% major and 21% minor discrepancy rates [29,30]. DMSVOs in particular are often difficult to detect in CT/CTA due to their distal location, smaller vessel diameter, tortuous course, and variable branching pattern [5,6]. The use of traditional axial, single-phase CTA is often challenging for the detection of M2 occlusions due to the small vessel caliber and the vessel course that does not run horizontal to the axial plane. In a large study conducted by Fasen et al. with 84 patients harboring large vessel occlusions, 20% (17/84) were missed in the initial CTA evaluation. However, on subset analysis, 82.4% (14/40) of the M2 segment occlusions were missed, which was a statistically significant difference compared with the occlusions of the distal internal carotid artery and/or the M1 segment (*p* = 0.01) [16].

Ospel et al. posited that multiphase CTA (mCTA) may be more sensitive in detecting DMSVOs in such scenarios [31]. mCTA is a modern imaging protocol consisting of two phases following the arterial phase in traditional CTA. It allows for the rapid determination and characterization of the collateral flow and the evaluation of the velocity of infarct progression, as a delay of contrast is often seen downstream in the correlating vascular territory [32]. It is less fraught with patient motion artifact than CTP, requires no additional contrast or radiation, and provides additional information regarding the true clot length, the detection of pseudo-occlusions, and distal clot assessment [33]. mCTA was successfully used in the ESCAPE and ESCAPE-NA1 trials and did not result in any imaging protocol violations and accurately characterized the infarcts and collateral flow without disrupting the workflow or needing any additional time [34,35].

At baseline, an mCTA display consists of three grayscale side-by-side images that must be linked together and scrolled through simultaneously, requiring an initial learning curve and familiarity for interpretation as opposed to the more visually friendly, single color-coded brain maps of CTP [36]. However, several studies have demonstrated the ability to incorporate vessel and blood flow information from all mCTA phases into a single color-coded display map similar to CTP, but with the inherent advantages of mCTA acquisition qualities, obviating the need for heterogenous image postprocessing algorithms and protocols [36,37]. In a study of 58 DMSVOs, detection using mCTA-derived tissue maps had a sensitivity of 91% (95% CI: 80–97) and a specificity of 82% (95% CI: 70–90) [38]. Further studies using mCTA combined with a novel deep convolutional neural network demonstrated a statistically significant improvement in the detection of large vessel occlusions, with 77% sensitivity and 71% specificity in single-phase CTA and 100% sensitivity and 77% specificity in mCTA (*p* = 0.01) [39]. Comparison studies between CTP and mCTA have also suggested that junior radiologists interpreted mCTA images with a high diagnostic accuracy in AIS, and furthermore, that mCTA demonstrated an increased predictive accuracy over CTP (*p* < 0.001) [40,41]. While single-phase CTA may suffice for PLVOs, occlusions of medium or small vessels are often missed, and mCTA is a tool that, with a wider implementation, may be used to improve the sensitivity in detection, especially in the ACA and PCA territories that are often not fully imaged with CTP [36].

Furthermore, artificial intelligence, in conjunction with mCTA, is an additional tool that has been investigated for use in detecting vessel occlusion [42]. In a study of 62 patients with LVO confirmed via catheter angiography, a novel deep learning model in conjunction with mCTA showed a statistically significant improvement in the ability to detect LVOs compared with single-phase CTA (AUC of 0.89, sensitivity of 100%, and specificity of 77% vs. AUC of 0.74, sensitivity of 77%, and specificity of 71%) [39]. This model of a neural network was built and trained on a dataset of 540 CTA scans (270 LVO-positive and 270 LVO-negative cases) that were collected retrospectively from the author’s institution. However, this model was only able to detect the global presence or absence of LVO without the classifiers of location or the detection of other intracranial pathologies, limiting its clinical utility as a triage tool. Furthermore, this study investigated the model’s utility in detecting LVOS and did not include DMSVOs. Testing this model in the setting of DMSVOs as well as further prospective data in the emergency room setting are needed to strengthen the potential clinical utility of using artificial intelligence and deep learning in conjunction with mCTA in detecting acute vessel occlusions [39].

The time-to-maximum (Tmax) is a parameter used in CTP, whereby the difference in time between the contrast arriving in the proximal vessel and the brain tissue is calculated via postprocessing, deconvoluting algorithms using the arterial input function [43]. In several large-scale clinical trials for PLVO, the Tmax has been utilized successfully for identifying remaining viable tissue following a vessel occlusion [44,45]. Its utility in the setting of a DMSVO is less studied but it may have even further utility, as DMSVOs are often more difficult to detect in single-phase CT due to their smaller caliber and more tortuous course, as well as the increased number and poorer opacification of distal arteries [46]. Studies have reported the sensitivity of the detection of DMSVOs to be as low as 33 percent, with 35% of M2 occlusions missed in the initial CTA evaluation [16,47,48]. However, when incorporating Tmax, one study found that using CTP-derived Tmax tissue residual function maps increased the sensitivity of DMSVO detection from 70.7 to 90.4 percent even when compared to mCTA, showing significant differences in the diagnostic accuracy, confidence, and speed [47].

Other advanced CTA imaging protocols were proposed to improve the detection of DMSVOs, including waveletCTA, ultra-high-resolution, and dual-energy CTA [17,49]. Wavelet-based CTA reconstruction in particular has been suggested to offer as high as a 9-fold increase in the signal-to-noise ratio compared to single-phase CTA, as well as an improved visualization of peripheral vessels [17,50]. One study of 59 patients with DMSVOs, utilizing wavelet-based CTA, found that occlusion was identified in 31 (52.5%) patients who originally had a negative single-phase CTA [17]. The authors described that wavelet CTA was able to detect occult occlusions, which are associated with larger cerebral blood flow defects, and that it may be useful for identifying more patients who are eligible for endovascular intervention. However, despite the CTA maps generated with this approach, the user still needs to localize the occlusion via interpreting complex vessel anatomy, and the need for specialized CT postprocessing as well as a lack of widespread availability limit the feasibility and implementation of this protocol [17,50].

## 5. Intervention Techniques

Multiple landmark trials have demonstrated the efficacy of mechanical thrombectomy for PLVOs, leading to significant improvements in the level of disability/dependence after stroke both within 6 h and less than 24 h from stroke onset [2,51]. Due to the success of MT in PLVOs, a natural next question has arisen regarding its efficacy in DMSVOs. With recent advancements in endovascular devices and methods, navigating longer, more tortuous, and smaller diameter vessels has become more feasible [13,19,20]. Largely, endovascular intervention for AIS consists of stent retriever thrombectomy, thromboaspiration, and/or thrombolysis.

### 5.1. Stent Retriever Thrombectomy and/or Thromboaspiration

Recent years have seen the advent and implementation of smaller devices with increased maneuverability that are capable of navigating the smaller vessel diameters and tortuous courses of DMSVOs [13,15]. The smaller vessel size places patients at an increased risk of perforation, vessel dissection, and vasospasm during endovascular procedures, thus necessitating smaller, more maneuverable devices to achieve safe recanalization [52,53]. Early stent retriever devices ranged in diameter from 4 to 6 mm, which limited their access to small- and medium-sized vessels. However, over the past several years, a new group of stent retriever devices (Trevo XP ProVue, Stryker, Freemont, CA, USA; Mindframe Capture LP, Medtronic, Minneapolis, MN, USA; pREset LITE, phenox GmbH, Bochum, Germany; Catch Mini, Balt, Montmorency, France; and Tigertriever 13, Rapid Medical, Yoqneam, Israel) with smaller diameters ranging from 2.5 to 3 mm was released [13,14,15].

Current techniques involve either the deployment of a mini stent retriever with exchange from a microcatheter to a distal low-profile aspiration catheter (3max, Penumbra), or the use of a long microcatheter (Headway Duo, Microvention, Aliso Viego, CA, USA) that is coaxial with a distal low profile aspiration catheter. In both cases, the stentriever and the distal low-profile suction catheter are pulled back inside a larger bore catheter placed in the M1, thus performing a SAVE to the Solumbra technique [54,55,56]. The purpose of this is to try to avoid pulling and straightening the M3–4 junction, especially after the Sylvian point, in order to reduce the risk of hemorrhage. Having a distal support catheter may theoretically reduce this risk.

The smallest of these stent retrievers, the Tigertriever 13, has a diameter of 2.5 mm and is compatible with 0.013-inch microcatheters (Table 1). Several studies have investigated its utility in treating DMSVO [13,57,58]. In a study of 17 patients with DMSVO (11 primary and 6 secondary cases following PLVO thrombectomy), Guengo et al. reported a 94% successful recanalization rate (mTICI ≥ 2b) with minimal mortality and morbidity, with 65% (11/17) of patients demonstrating a favorable mRS at 3 months [13]. Only three patients had a subsequent vasospasm, and one patient had a minor procedural hemorrhage, which was not symptomatic. Interestingly, this study used the Tigertriever 13 stent in a wide variety of both anterior and posterior circulation DMSVOs, demonstrating the adaptability as well as the safety and feasibility of using this low-profile stent retriever in the treatment of DMSVO [13]. Etter et al. used the 3 and 4 mm Trevo NXT Provue Retriever (Stryker, Freemont, CA, USA) in 22 patients with M2 occlusion and achieved a 100% final reperfusion rate mTICI ≥ 2b (86% mTICI ≥ 2c, 59% mTICI 3), with an average pass number of 1 [59]. The authors reported only two cases of vasospasm and no cases of symptomatic intracranial hemorrhage. Despite being originally developed for use in PLVO, this mini stent retriever showed similar or better rates of recanalization in this study when used in M2 occlusions (22/22, 100%) compared with cases of PLVO (M1 or ICA-terminus, 49/52, 94%) and, interestingly, required less passes on average (1.0 passes for DMSVO and 1.5 passes for PLVO) [59]. Although further multicenter, randomized studies are warranted to further support the efficacy of using mini stent retrievers for DMSVOs, this new generation of mini-stent retrievers appears, in early studies, to be safe and feasible for a variety of vessel sizes due to its adaptable, low-profile design and maneuverability.

Catheter thromboaspiration alone or in conjunction with a stent retriever has also been used in the treatment of both PLVO and, more recently, DMSVO [60,61] (Table 1). Aspiration thrombectomy, as opposed to stent retriever thrombectomy, confers benefits of both time and cost and further creates a conduit for the rescue use of a stent retriever in the case of aspiration failure [62]. In a study of 270 patients with PLVO, first-line thromboaspiration was shown to be noninferior to a stent retriever in the rates of recanalization, hemorrhage, and functional outcome at 90 days, suggesting that this technique is a safe and feasible alternative to stent retriever use [63]. Its utility in DMSVOs, however, is less studied. One large study compared the use of mini stent retrievers alone with the blind exchange/mini-pinning technique (BEMP; a mini stent retriever combined with a low-profile 0.035-inch distal aspiration catheter) in the setting of a DMSVO [19]. In a series of 106 DMSVOs, the BEMP technique demonstrated significantly increased rates of first-pass recanalization (57% versus 34%, *p* = 0.017) as well as lower rates of symptomatic intracranial hemorrhage, new emboli, and needing rescue therapy. Following a multivariate analysis, the one independent variable that was significantly associated with first-pass expanded thrombolysis in cerebral ischemia 2c/3 recanalization was the use of the BEMP technique (odds ratio, 2.72 [95% CI, 1.19–6.22]; *p* = 0.018). However, there is a further need for large-scale prospective randomized controlled trial data to further support the use of these techniques in DMSVO [19].

**Table 1:** Selected stentriever and/or thromboaspiration studies for DMSVO.

	Device	Recanalization Rate (mTICI ≥ 2b)	Complication Rate (Vasospasm; Procedural Bleed)	% Patients with 90-Day mRS 0–2
Guengo et al. [13]	Tigertriever 13	94% (16/17)	23% (3; 1)	65 (11/17)
Kurre et al. [14]	pReset Lite	70% (63/90)	9% * (5; 0)	33 (25/76)
Etter et al. [59]	Trevor NXT Provue (CA)	100% (22/22)	9% (2; 0)	^‡^
Vega et al. [15]	Catchview Mini	91% (41/45)	18% (0; 8)	58 (26/45)
Perez-Garcia et al. [19]	Stentriever alone	78% (39/50)	10% (^†^; 5)	53 (26/49)
	BEMP	79% (44/56)	2% (^†^; 1)	51 (27/53)
Atchaneeyasakul et al. [64]	Stentriever alone	90% (108/120)	3% (^†^; 4)	52 (63/120)
	Aspiration alone	77% (60/77)	17% (^†^; 13)	37 (28/77)

Abbreviations: distal medium and small vessel occlusion (DMSVO); symptomatic intracranial hemorrhage (sIHC); combined approach (CA); blind exchange/mini-pinning technique (BEMP); * 2 suspected dissections, 1 self-limiting extravasation, 5 cases of vasospasm; ^†^ data not reported in study; ^‡^ not reported; median NIHSS values 24 h post procedure were 4.5 (IQR, 1.5–11) and 2 (IQR, 0–5) at discharge.

### 5.2. Indications

As of now, there are no clear, definite indications for endovascular intervention in DMSVO, but current clinical trials have intervened in cases of significant neurological deficit (hemianopsia, motor, and aphasia) or NIHSS > 4, with a mismatch of 1 or more on CTP. Table 2 lists the major currently ongoing clinical trials for DMSVO as well as their primary outcomes, interventions, and inclusion criteria.

### 5.3. Intravenous/Intra-Arterial Fibrinolysis

Intravenous thrombolysis with a tissue plasminogen activator (tPA) or other fibrinolytics has long been a standard of care in AIS, facilitating the reperfusion of healthy brain tissue and the preservation of functional status [65]. Reports have described an increased efficacy of intravascular fibrinolytics in DMSVOs relative to PLVOs, which is thought to be the result of the overall difference in the clot burden [66]. Yoo et al. demonstrated, in a study including both retrospective and prospective cohorts (214 and 78 patients, respectively), that in the patients who were treated with IV tPA who failed to achieve recanalization, the thrombus volume was significantly larger than in those who achieved successful recanalization (149.5 ± 127.6 versus 65.3 ± 58.3 mm^3^; *p* < 0.001), and the thrombus volume was an independent predictor of recanalization [66].

In contrast to the traditional intravenous route, intra-arterial thrombolysis has also been investigated and shown to be safely administered to patients with acute stroke onset of less than 6 h in the MCA territory [67]. This facilitates a higher concentration of tPA to the thrombus, allows for access to a wide range of brain regions as well as all major intracranial vessels, and potentially reduces systemic lytic exposure and overall drug concentrations [68]. Many agents have been trialed including rTPA, desmoteplase, tenecteplase, streptokinase, pro-urokinase, reteplase, plasmin, staphylokinase, and microplasmin [69]. Tenecteplase possesses a longer plasma half-life, greater fibrin specificity, and an improved benefit-to-risk ratio than alteplase, and thus has been suggested for use in patients with acute myocardial infarction [70]. Two large randomized trials demonstrated similar clinical outcomes between patients treated with alteplase versus tenecteplase in acute ischemic stroke [71,72]. In the ACT trial, 304/801 and 279/757 patients treated with tenecteplase versus alteplase, respectively, harbored DMSVOs. Overall, there was no significant difference in the percentage of patients with mRS < 2 at 90 days between the two treatment groups [71]. However, the results from the EXTEND-IA TNK clinical trial suggest that the administration of tenecteplase before MT resulted in higher reperfusion rates and improved the 90-day mRS compared to alteplase [73]. A further subgroup analysis from this trial suggested that tenecteplase was superior to alteplase in distal M1 and M2 occlusions (53/176 vs. 4/42) as well as in lesions with a low clot burden, and that efficacy was limited in cases of internal carotid artery occlusion or high clot burden [74]. However, dedicated multicenter randomized controlled trials investigating the long-term efficacy of tenecteplase versus alteplase for DMSVO are still lacking and are warranted to optimize treatment guidelines.

The PROACT-2 trial demonstrated the safety and efficacy of intra-arterial prourokinase in patients with acute ischemic stroke of less than 6 h in the M1 or M2 MCA (including 44 patients with isolated M2 occlusions), with a recanalization rate of 66% for the treatment group versus 18% for the group receiving heparin only [67,75]. The number of patients with mRS < 2 at 90 days was significantly less in the treatment arm (121 vs. 59, respectively; OR 2.13 (95% CI: 1.02–4.42); *p* = 0.04) despite there being a slightly increased risk of early symptomatic intracerebral hemorrhage [67].

Despite the recent advances in endovascular device design for MT in DMSVOs, widespread use has yet to be adopted due to limiting factors including device availability, vessel size/tortuosity, as well as operator skill and the learning curve [20]. A large multicenter survey of 366 neurologists, interventional neuroradiologists, and neurosurgeons revealed that neurointerventionalists were willing and inclined to treat DMSVO promptly with MT if feasible [76]. However, it was also shown that treatment with IV tPA was significantly negatively associated with proceduralist decision making to proceed directly with MT, although the majority of physicians ultimately decided to proceed with MT without waiting for an alteplase treatment effect [77].

Although intravascular thrombolysis has demonstrated safety and efficacy in DMSVOs and may be more beneficial than in cases of PLVOs, between one-half and two-thirds of occlusions still fail to recanalize following therapy [78,79]. Investigators have further raised the question of combining intravenous thrombolysis with MT. A large-scale clinical trial demonstrated significantly increased efficacy and improved outcomes of thrombectomy within 6 h after receiving IV tPA versus tPA alone [52]. Subsequently, the results of the multicenter SKIP trial showed no significant difference between those treated with alteplase plus MT versus MT alone [80]. To date, there have been no dedicated large-scale multicenter clinical trials investigating the efficacy of MT versus thrombolysis or a combination of the two in DMSVOs while utilizing newly available, innovative devices that are capable of treating pathology in distal vessels. Future studies are warranted to evaluate the safety and efficacy of these new methods in treating DMSVOs as well as to characterize the risk and benefit profiles.

## 6. Summary and Management Algorithm

The current guidelines for the management of acute ischemic stroke, such as those described in the European Stroke Organisation, incorporate data from large-scale multicenter randomized clinical trials to provide management recommendations for PLVO. In cases of acute ischemic stroke due to an anterior circulation PLVO presenting to a capable treatment center within 4.5 h of symptom onset, intravenous thrombolysis plus MT is recommended versus MT alone [21]. Furthermore, the data from the DEFUSE-3 trial suggest that thrombectomy plus medical treatment 6 to 16 h following symptom onset resulted in superior functional outcomes compared to standard medical therapy alone in PLVO [51]. The results from the DAWN trial further expanded this window and suggested that MT plus medical therapy in cases of PLVO within 24 h of the last known well resulted in a significantly improved mRS value at 90 days compared with standard care alone [2]. However, large-scale prospective randomized clinical trials for DMSVO are limited compared to those for PLVO.

**Figure 1:**
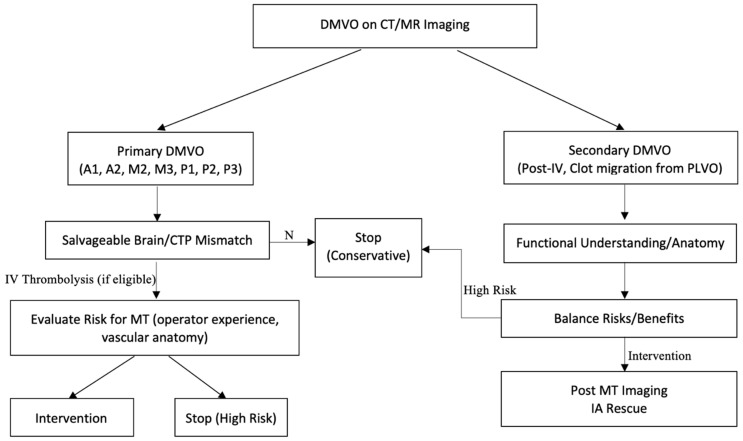
Proposed management algorithm for detection and intervention of DMSVOs.

When patient symptoms and radiographic evidence of acute ischemic stroke are present, it is first important to consider the clinical context and determine the etiology of the DMSVO (Figure 1). In the case of symptom onset following intravenous intervention or in the setting of a prior known PLVO, the suspicion for a secondary DMSVO should be high as a result of clot migration. In such cases, the location, the functional understanding of the anatomy and vascular territory, as well as the risks and benefits regarding the baseline functional status should be considered before making the ultimate decision to pursue MT. In the case of a primary DMSVO (A1, P1, A2, M2, M3, P2, and P3) and a pertinent deficit in a correlating vascular territory, CTP should be used to evaluate perfusion mismatch and determine the amount of viable brain tissue. If the stroke is completed with minimal viable neural tissue in CTP as well as a low patient functional status, supportive measures should be pursued without additional aggressive intervention. In the case of CTP mismatch and significant salvageable brain tissue (i.e., penumbra), IV thrombolysis may be pursued within the appropriate window before evaluating the risks of MT (operator experience, difficulty of vessel anatomy, etc.). If the procedural risks/benefits are considered and the decision to pursue MT is made, post-MT imaging within 24 h is critical in the case of neurological worsening as well as to evaluate the arterial patency, the success of recanalization (mTICI score), the risk of hemorrhagic transformation, the final infarct volume, and any need for intra-arterial rescue therapy.

## 7. Limitations

This literature review was limited in its retrospective nature and due to the lack of randomized prospective data. Therefore, primary data and follow-up are not directly available from this study. Furthermore, many of the studies referencing the use of modern techniques for DMVO are single-institution experiences with a limited number of patients. For both the imaging and intervention of DMSVO, each of these methods (i.e., mini stentrievers, mCTA, etc.) warrants additional studies and more data points. While initial safety and feasibility may be suggested from such smaller studies, the efficacy and long-term safety need to be established in larger, prospective, randomized cohorts of patients. Furthermore, as this literature review does not provide any additional patients, we did not perform any statistical analyses of our own in the evaluation of the described methods. However, the goal of this study was to provide a description and an update of these imaging and interventional methods. In order to evaluate the efficacy and long-term safety, future large-scale multicenter randomized controlled trials with larger numbers of patients are warranted.

## 8. Conclusions

Early studies have suggested that mechanical thrombectomy may be a safe and feasible treatment modality for DMSVO. Using modern methods for both the imaging (i.e., mCTA) and treatment (mini stentrievers ± thromboaspiration, thrombolysis, etc.) of DMSVO may allow for more sensitivity, specific detection, and therapeutic intervention for vessel occlusions of smaller and more tortuous vessels. However, further large-scale prospective randomized controlled trials are needed to further solidify the safety and efficacy of these methods as well as to refine the procedural indications. Further improvements in imaging, neuro-critical care, and small maneuverable devices will likely allow for neurointerventionalists to expand the indications for the treatment of DMSVO.

## Figures and Tables

**Table 2:** Current ongoing major clinical trials for DMSVO.

	DISCOUNT (NCT05030142)	DISTALS (NCT05152524)	ESCAPE-MeVO (NCT05151172)	DISTAL (NCT05029414)
Primary Outcome	mRS 0–2 at 90 days	Successful reperfusion (CT or MR PWI *) without sICH	mRS at 90 days	mRS at 90 days
Material	Trevor NXT Provue Catchview mini pReset Lite Tigertriever13	Tigertriever 13	Solitaire X	All commercially available CE-certified revascularization devices (stentrievers, thromboaspiration/balloon guide catheters)
Main Inclusion Criteria	NIHSS ≥ 5; -Distal M2 (above mid-height of insula) -M3, P1, P2, or P3 -A1, A2, or A3	NIHSS 4–24 or NIHSS 2–24 if aphasia and/or hemianopia; -ACA; non-dominant or co-dominant M2; M3; PCA -Vessel ≥ 1.5 mm -Ischemic core in ≤50% of perfusion lesion volume	NIHSS ≥ 5 or 3–5 (if disabling symptoms); -M2 or M3, A2 or A3, P2 or P3	NIHSS ≥ 5 or “clearly disabling symptoms” -co-/non-dominant M2, M3/M4, A1/A2/A3, P1/P2/P3d

Abbreviations: Modified Rankin Scale (mRS); Magnetic Resonance Perfusion Weighted Imaging (MR PWI); National Institutes of Health Stroke Scale (NIHSS); Evaluation of Mechanical Thrombectomy in Acute Ischemic Stroke Related to a Distal Arterial Occlusion (DISCOUNT); Distal Ischemic Stroke Treatment with Adjustable Low-Profile Stentriever (DISTALS); Endovascular Treatment to Improve Outcomes for Medium Vessel Occlusions (ESCAPE-MeVO); Middle Cerebral Artery M3 Segment (M3); Posterior Cerebral Artery Segments (P1, P2, P3); Anterior Cerebral Artery Segments (A1, A2, A3); Endovascular Therapy plus Best Medical Treatment (BMT) versus BMT Alone for Medium Vessel Occlusion Stroke (DISTAL). * >50% reduction in Tmax > 4 s within 24 ± 6 h.

## Data Availability

Not applicable.
